# Autonomy support in prenatal consultation: A quantitative observation study in maternity care

**DOI:** 10.18332/ejm/197053

**Published:** 2025-01-13

**Authors:** Joyce Kors, Linda Martin, Corine J. Verhoeven, Jens Henrichs, Saskia M. Peerdeman, Rashmi A. Kusurkar

**Affiliations:** 1 Amsterdam UMC location Vrije Universiteit Amsterdam, Research in Education, Amsterdam, Netherlands; 2 LEARN! Research Institute for Learning and Education, Faculty of Psychology and Education, Vrije Universiteit Amsterdam, Amsterdam, Netherlands; 3 Amsterdam UMC location Vrije Universiteit Amsterdam, Department of Midwifery Science, AVAG, Amsterdam Public Health Research Institute, Amsterdam, Netherlands; 4 Department of Obstetrics and Gynecology, Maxima Medical Centre, Veldhoven, Netherlands; 5 Division of Midwifery, School of Health Sciences, University of Nottingham, Nottingham, United Kingdom; 6 Amsterdam UMC, Faculty of Medicine, VU University Amsterdam, Netherlands; 7 Amsterdam Public Health, Program Quality of Care, Amsterdam, Netherlands

**Keywords:** self-determination theory, prenatal consultations, autonomy-supportive consultation

## Abstract

**INTRODUCTION:**

Maternity care professionals need to guide women through an increasing number of decision-making processes during pregnancy. Professionals tend to focus more on providing information than on decision support. According to the self-determination theory (SDT), professionals could help women make their own choices by fulfilling their three basic psychological needs: autonomy, competence, and relatedness through autonomy-supportive interactions. This study aimed to quantify autonomy-supportive and autonomy-thwarting interactions that professionals use during prenatal consultations and their association with women’s perceptions of the healthcare climate during consultations.

**METHODS:**

A quantitative observation study with a cross-sectional design was conducted in the Netherlands from March to October 2020. Twenty-three maternity care professionals in 2 hospitals and 16 midwifery practices were purposefully sampled. During 104 prenatal consultations, professional interactions were audiotaped and coded using the Coding and Observing Need-Supportive Consultation in Maternity Care Consultations. The woman's perceived healthcare climate was assessed using the Healthcare Climate Questionnaire.

**RESULTS:**

We observed that professionals derive their autonomy-supportive interactions from a small repertoire. They tend to use more autonomy-supportive interactions (mean=2.31, SD=0.58) that give room to the woman than interactions that stimulate active engagement (mean=1.41, SD=0.80). During structuring interactions, they tend to use more informative (mean=1.81, SD=0.59) than supportive interactions (mean=0.94, SD=0.55). Women generally perceived the healthcare climate as positive.

**CONCLUSIONS:**

Women were rarely stimulated to be actively engaged in the consultations, while active woman engagement is vital in offering women-centered decision-making support. Professionals could improve their autonomy-supportive consultation climate by paying explicit attention to interactions involving women and offering structure.

## INTRODUCTION

Maternity care professionals need to guide women through an increasing number of decision-making processes during prenatal consultations. The number of topics that need to be discussed and the number of decisions that need to be made in prenatal consultations have increased due to the broader scope of screening for genetic disorders, the increasing number of options for care during pregnancy and birth, and the trend to start supporting the transition into parenthood during pregnancy^1^.

Professionals find it challenging to guide women through these decision-making processes. Research has shown that despite the attention to decision-making in professional training, professionals still tend to offer decision support mainly by giving information^2-6^. Although providing information is part of the decision-making process, women need more support to make their decisions, such as encouragement to think about a possible decision^2,7^. Research in maternity care shows that professionals dedicate much effort to building and protecting their relationship with their women^8^. However, in these conversations, there is little room to discuss women’s fears, values and expectations, which is necessary for decision support^3,7^, especially if decisions need to be reconsidered in light of changing circumstances.

**Figure 1:**
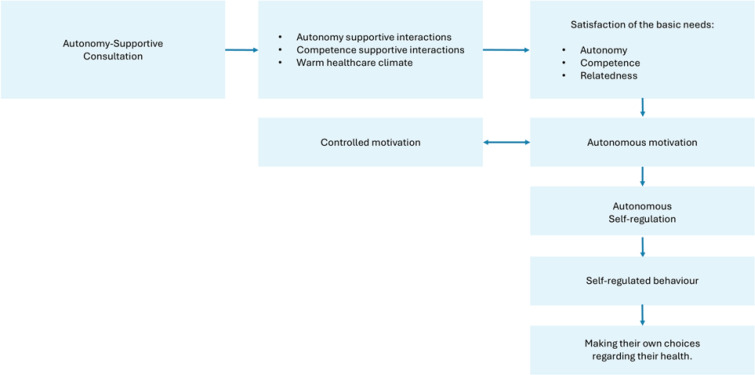
Model of autonomy-supportive consultation, quantitative observation study in maternity care in the Netherlands, 2024

The self-determination theory (SDT), a macro theory of human motivation, states that people become more autonomously motivated when their basic psychological needs of autonomy (feeling of ownership, endorsement, and choice), competence (feeling of capability and growth), and relatedness (feeling of belonging and connection) are met. In autonomy-supportive consultation (ASC), professionals create an autonomy-supportive healthcare climate using an autonomy-supportive interaction style to meet patients’ basic psychological needs. In doing so, they facilitate more autonomous forms of motivation, resulting in more self-regulating behavior in patients, which enables them to make their own choices (Figure 1)^9,10^.

**Figure 2:**
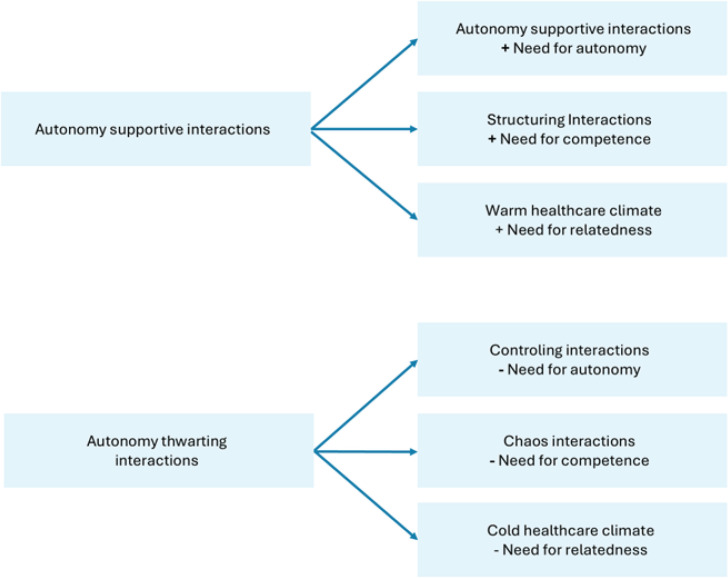
Autonomy-supportive and autonomy-thwarting interactions, quantitative observation study in maternity care in the Netherlands, 2024

Healthcare professionals can use two types of interactions: autonomy-supportive interactions that satisfy women’s basic psychological needs, and autonomy-thwarting interactions that frustrate these needs. Regarding autonomy-supportive interactions, professionals can meet women’s need for autonomy by using interactions, such as listening reflectively and exploring women’s thoughts. They can meet women’s need for competence by structuring interactions, such as providing information and asking women to summarize and request repetition. Autonomy-thwarting interactions include controlling interactions, which frustrate women’s need for autonomy, and chaotic interactions, which frustrate their need for competence. Women’s need for relatedness can be satisfied by creating a warm healthcare climate, for example, by using emphatic listening, whereas a cold healthcare climate thwarts women’s need for relatedness (Figure 2).

This study aimed to quantify autonomy-supportive and autonomy-thwarting interactions that maternity care professionals use in daily practice. In addition, we aimed to establish whether there is an association between autonomy-supportive and autonomy-thwarting interactions and the characteristics of women and professionals and whether there is an association between these interactions and the woman-perceived healthcare climate during consultations.

To reach our aim, four research questions were formulated. First, we will investigate which autonomy-supportive and autonomy-thwarting interactions maternity care professionals use in prenatal consultation in daily practice and the frequency of these interactions. Second, we will examine which characteristics of women or professionals are associated with using autonomy-supportive or autonomy-thwarting interactions. Next, we investigate if there is a relation between autonomy-supportive or autonomy-thwarting interactions and maternity care professional characteristics. Finally, we investigate whether there is a relation between autonomysupportive or autonomy-thwarting interactions and the woman-perceived healthcare climate during consultations.

## METHODS

### Study design and setting

This quantitative observational study with a cross-sectional design was conducted in the Netherlands. Quantitative data were collected and analyzed via a structured observation approach to answer the research questions and explore the professional–woman interaction^11^. Recruitment started in January 2020, and data collection occurred from March to October 2020, with two interruptions due to a COVID-19 lockdown. The study occurred during prenatal consultations in maternity care in an academic hospital, a teaching hospital, and 16 small and large midwifery practices in urban and rural areas across the country.

### Participants

We purposefully sampled professionals from a wide variety of settings in which the prenatal consultations took place via the Childbirth Network and our hospital^12^. Because almost 90% of the women start their prenatal care in primary care in the Netherlands, we included more midwifery practices than hospitals^13^. The women were included if they had an appointment for a prenatal consultation during the planned observation period as per the convenience of the first author (JK). Thus, the women were not selected a priori.

All women with appointments during the observation period received written information about the study from their maternity care professional. The first author (JK) requested the informed consent of both the professional and the woman. If informed consent was obtained, the first author attended the consultation and audiotaped the interaction. In line with earlier quantitative observational studies in this field, we assumed that including a minimum of 20 professionals for observation during five consultations per professional would be enough^14^. Concerning the number of possible observed consultations, this sample size is higher than the minimally required sample of 50 participants for testing correlations^15^.

### Data sources and measurement

During the included consultations, various issues were discussed, and decisions were made on participation in prenatal screening or an immunization program, preferred care during birth, an exercise program or women’s diet.

The interactions during the consultation were observed and coded using the Coding and Observing Need-Supportive Consultation in Maternity Care Consultations (CONSUL-MCC). This adapted SDT-based observation tool has been validated for use in maternity care^16^. The observation tool facilitates the observation and coding by defining the various aspects of the care professional’s performance and by instructing the assessors on what to focus on and how to judge their observations^17^.

The CONSUL-MCC comprises a manual describing and illustrating each item by an example (Supplementary file Table 1). The manual also includes instructions on assessing the audio fragments and encoding the items using a score form. The audiotaped consultations were divided into units of 5 minutes each to facilitate the assessors’ focus on encoding each fragment on all 41 items. Each item was scored on a 5-point Likert scale. The coding ranged from 0 (not observed at all) to 4 (observed continuously).

The CONSUL-MCC encodes the interactions of maternity care professionals on two axes: autonomy-supportive versus control, and structure versus chaos. Each factor is divided into two subfactors, and each subfactor is operationalized using three to six observable items (Supplementary file Figure 1). The score per item, mean scores per subfactor and factor could be calculated. The healthcare climate has two overall items: the extent to which the climate is observed as warm and the extent to which the climate is observed as cold. A warm climate means that the professional is attentive to the woman’s responses and uses emphatic listening, acknowledges the woman’s expressed feelings and emotions, engages in warm and friendly communication and shows unconditional respect, regardless of the woman’s behavior. The climate is considered cold if the professional fails to establish connectivity and reciprocity^18^.

JK observed and encoded the consultations for the first time in daily practice to gain a general impression. Afterwards, the 5-minute units were encoded. To improve reliability, the coding of the 5-minute fragments was compared to the coding in daily practice. In case of discrepancies, the audiotaped consultation was reassessed.

After each consultation, the woman was asked to complete the Dutch version of the Healthcare Climate Questionnaire (HCCQ). This questionnaire was translated into Dutch and validated for use in Dutch mental healthcare by Jochems^19,20^. For use in maternity care, we replaced ‘the practitioner’ with ‘the midwife/obstetrician’. We substituted the examples from the context of mental health with examples from the context of maternity care. The reliability of the adapted HCCQ was measured by calculating the Cronbach alfa. The Cronbach’s alfa of the adapted HCCQ was 0.64, which was considered acceptable^21^. The adapted HCCQ comprises 15 statements, and women score their level of agreement on a 7-point Likert scale (1=strongly disagree to 7=strongly agree) (Supplementary file Table 2). The mean score per statement and the total mean score over all statements could be calculated.

The following characteristics of women were collected: age, parity, and education level. Characteristics collected from the professionals were age and work experience.

### Ethical considerations

The Medical Ethics Review Committee (METc) of Amsterdam UMC stated that this research is not subject to the Medical Scientific Research with Humans Act (WMO) (case number 2019.415). All participants received an information letter and a verbal explanation of the research and the audio-taping. Participation was voluntary. The participating maternity care professionals and women provided their written consent.

### Statistical analyses

First, we analyzed the characteristics of the participants and the consultations using descriptive statistics (mean, SD and range) in the case of continuous variables. Demographic categorical characteristics were expressed in absolute numbers and percentages. To assess the frequencies of the interactions professionals use during their prenatal consultations and the observed healthcare climate, which were expressed as continuous variables, we used descriptive statistics at the consultation level, i.e. for the entire consultation. A visual inspection of the frequency distribution (using histograms) of the various interaction and healthcare climate variables revealed non-normality in all variables^22^. Therefore, we report medians, modes and ranges for these variables.

Because of the non-normal distribution of the data in the current study, we used non-parametric tests for our main analysis^22^. That is, we computed Spearman’s correlations to investigate the correlation between the women’s age, parity or education level, and the level of autonomy support or structure, and between the professionals’ age or work experience and the level of autonomy support or structure. Moreover, we examined the differences in levels of autonomy support interactions and structuring interactions across the various individual professionals using the independent-samples Kruskal-Wallis test again due to the non-normality of the outcome variables mentioned above.

To assess the woman-perceived healthcare climate, we analyzed the absolute and relative frequencies (percentages) of the level of agreement for each statement. We computed Spearman’s correlations to find correlations between professionals’ interactions and the non-normally distributed healthcare climate scores as reported by women. Using Spearman’s correlations, we also investigated if autonomy-supportive or structuring interactions were correlated with the woman-perceived healthcare climate. We then assessed the differences in levels of the healthcare climate as perceived by the women across the various individual professionals using the independent samples Kruskal-Wallis test. For all quantitative analyses complete case analysis was applied. All quantitative analyses were conducted using IBM SPSS 28. A significance threshold of p<0.05 was used.

## RESULTS

### Data characteristics

*Maternity care professionals*

Of the 23 maternity care professionals who participated in this study, 17 were primary care midwives, 2 were hospital-based midwives, and 2 were obstetricians. The mean age of these participants was 38 years (range: 25–64 years), and their mean work experience was 14 years (range: 3–43 years).

*Women*

In total,104 women participated in this study. Their average age was 32.5 years (range: 21–44 years). Of these women, 34 (32.7%) were pregnant for the first time, 41 (39.4%) for the second time and 29 (27.9%) for the third time or more (3–6). The education level distribution was: lower secondary, 4.8%; upper secondary, 3.9%; secondary vocational, 26.9%; university of applied science, 35.6%; and university, 28.8%.

*Consultations*

In total, 104 consultations were observed. The mean consultation duration was 21 minutes (range: 7–73 minutes), resulting in 453 five-minute fragments. Most of the longer consultations included an ultrasound scan.

### Autonomy-supportive and autonomy-thwarting interactions of maternity care professionals

Table 1 shows the frequencies of the interaction items covering: autonomy support versus control, and structure versus chaos. In general, the professionals used interactions based on a small repertoire. We found that professionals regularly use autonomy-supportive interactions, such as ‘leaves room to the woman to tell’ (mean=2.34, SD=0.57), and they sometimes use structuring interactions, such as ‘clarifies follow-up of woman’s goals’ (mean=1.77, SD=0.55). They rarely used controlling interactions (mean=0.73, SD=0.46) and hardly ever used chaotic interactions (mean=0.09, SD=0.11). The most frequently used controlling interaction was ‘determines the topics of the conversation’ (mean=1.19, SD=0.96). In the further description of the results, we focus on the autonomy-supportive and structuring interactions.

**Table 1:** Observed autonomy-supportive and autonomy-thwarting interactions, quantitative observation study in maternity care in the Netherlands among 23 maternity care professionals during 104 prenatal consultations, 2020

		*Mean*	*SD*	*Min*	*Max*	*Median*	*Mode*
***Autonomy support***		2.34	0.57	0.50	3.00		
**Attuning approach**		2.31	0.58	0.50	3.00		
1	Leaves room to the woman to tell	2.43	0.57	1.00	3.00	2.60	3.00
2	Listens reflectively and explores	1.03	0.75	0.00	3.00	0.83	0.00
3	Aligns with the woman’s perspective	1.93	0.92	0.00	3.00	2.00	3.00
4	Uses questions that offer space to the woman	1.75	0.74	0.25	3.00	1.67	2.00
5	Allows emotions and actively names them	0.28	0.41	0.00	2.00	0.00	0.00
6	Uses inviting language	2.31	0.69	0.40	3.00	2.33	3.00
**Participative approach**		1.41	0.80	0.00	3.00		
7	Allows time	0.65	0.70	0.00	2.67	0.27	0.00
8	Gives voice	0.95	0.69	0.00	3.00	0.75	0.50
9	Explores the woman’s goals	0.85	0.64	0.00	2.33	0.75	0.00
10	Encourages to think about a possible approach	0.78	0.72	0.00	3.00	0.67	0.00
10a	Encourages to think about a possible approach with important kin	0.06	0.17	0.00	1.00	0.00	0.00
11	Offers an explanation	1.25	0.99	0.00	3.67	1.00	0.00
12	Actively gauges what degree of autonomy the woman wants	0.04	0.029	0.00	0.21	0.00	0.00
***Control***		0.73	0.46	0.00	1.75		
**Demanding approach**		0.72	0.46	0.00	1.75		
13	Shows expertise, demands respect	0.019	0.09	0.00	0.67	0.00	0.00
14	Uses controlling language	0.16	0.33	0.00	2.00	0.00	0.00
15	Takes over the conversation	0.18	0.31	0.00	1.50	0.00	0.00
16	Determines the topics of the conversation	1.19	0.96	0.00	3.00	1.00	1.00
17	Interrupts the woman	0.13	0.30	0.00	2.25	0.00	0.00
**Domineering approach**		0.03	0.11	0.00	0.80		
18	Puts pressure on the woman	0.02	0.06	0.00	0.40	0.00	0.00
19	Expresses criticism	0.02	0.07	0.00	0.50	0.00	0.00
20	Introduces guilt and shame	0.00	0.00	0.00	0.00	0.00	0.00
21	Is irritated in woman	0.02	0.10	0.00	0.82	0.00	0.00
***Structure***		1.77	0.55	0.64	3.00		
**Guiding approach**		0.94	0.71	0.00	3.00		
22	Sets realistic goals in collaboration	0.49	0.56	0.00	3.00	0.33	0.00
23	Provides task-oriented or progress-oriented feedback	1.36	0.88/ 0.89	0.00	3.00	1.21	1.00
24	Stimulates self-reflection	0.13	0.25	0.00	1.00	0.00	0.00
25	Mentions previous successes	0.07	0.18	0.00	1.00	0.00	0.00
26	Uses tools	0.39	0.40	0.00	2.50	0.33	0.00
**Clarifying approach**		1.81	0.59	0.64	3.00		
27	Provides alternatives	0.25	0.43	0.00	2.40	0.00	0.00
28	Uses appropriate role models	0.03	0.12	0.00	0.67	0.00	0.00
29	Provides information	2.41	0.66	0.33	3.50	2.50	3.00
30	Summarizes and requests repetition	0.18	0.24	0.00	1.00	0.00	0.00
31	Clarifies follow-up of woman’s goals	0.92	0.58	0.00	3.00	1.00	1.00
32	Uses attuned language	3.00	0.07	2.50	3.33	3.00	3.00
***Chaos***		0.09	0.19	0.00	0.80		
**Abandoning approach**		0.08	0.21	0.00	1.29		
33	Provides information that leaves woman in uncertainty	0.07	0.17	0.00	1.00	0.00	0.00
34	Gives inappropriate feedback	0.02	0.09	0.00	0.71	0.00	0.00
35	Uses an illogical conversation structure	0.004	0.03	0.00	0.25	0.00	0.00
36	Ignores reactions or concerns	0.05	0.18	0.00	1.00	0.00	0.00
**Awaiting approach**		0.05	0.14	0.00	0.67		
37	Does not intervene	0.008	0.008	0.00	0.08	0.00	0.00
38	Lets the woman find out for herself	0.02	0.09	0.00	0.67	0.00	0.00
39	Is distracted, absent	0.05	0.16	0.00	1.00	0.00	0.00
**Healthcare climate**							
40	Warm	2.76	0.39	1.80	3.33	3.00	3.00
41	Cold	0.004	0.03	0.00	0.25	0.00	0.00

The contributions of the sub-factors to each factor differ. Within the factor ‘autonomy support’, professionals more frequently used an attuning approach, starting from the woman’s perspective (mean=2.31, SD=0.58), than a participatory approach, placing the woman’s agenda centrally and providing direction together with the woman (mean=1.41, SD=0.80). Furthermore, within the factor ‘structure’, professionals more frequently used a clarifying approach, providing information to the woman (mean=1.81, SD=0.59) than a guiding approach, providing support that makes the woman more competent (mean=0.94, SD=0.55).

There was a wide variation between the interaction items belonging to the sub-factors (attuning, participative, guiding and clarifying). The following interaction items were observed frequently: ‘leaves room to the woman to tell’ (mean=2.43, SD=0.57), ‘uses inviting language’ (mean=2.31, SD=0.68), ‘provides information’ (mean=2.41, SD=0.66) and ‘uses attuned language’ (mean=3.00, SD= 0.07). Other items were rarely observed: ‘allows emotions and actively names them’ (mean=0.28, SD=0.41), ‘encourages to think about a possible approach with important kin’ (mean=0.06, SD=0.17), ‘actively gauges what degree of autonomy the woman wants’ (mean=0.04, SD=0.03), ‘stimulates self-reflection’ (mean=0.13, SD=0.25), ‘mentions previous successes’ (mean=0.07, SD=0.18), ‘provides alternatives’ (mean=0.25, SD= 0.43), ‘uses appropriate role models’ (mean=0.03, SD=0.12) and ‘summarizes and requests repetition’ (mean=0.18, SD=0.24) (Table1).

### Association between professionals’ and women’s characteristics and autonomy-supportive interactions

There were no statistically significant associations between the woman’s age, parity, education level and the level of autonomy support (r=0.041, p=0.68; r=0.072, p=0.47; r=0.032, p=0.75; respectively) or structure (r= -0.119, p=0.23; r= -0.131, p=0.18; r= -0.061, p=0.54; respectively).

There were no statistically significant associations between the professionals’ age or work experience and the level of autonomy support (r=0.018, p=0.86; r=0.032, p=0.75; respectively) or structure (r=0.081, p=0.42; r=0.109, p=0.27; respectively).

Levels of autonomy-supportive or structuring interactions significantly differed between the individual professionals (p<0.001).

### Woman-perceived healthcare climate

Women generally perceived the healthcare climate during prenatal consultations as positive, as shown in Table 2. Their mean score on a 7-point Likert scale was above 6.29 for 14 of the 15 statements (6.29–6.95). Only statement 11, about how maternity care professionals deal with the woman’s emotions, scored lower (3.78). According to 44.2% of the women, dealing with emotions was inapplicable during their consultation. Looking more closely, despite the small numbers, there was a little more spread on the statements which refer to active woman participation. This was also the case for the three statements, which refer to the extent to which the professionals take the uniqueness of their women into account.

**Table 2:** Descriptive statistics of the woman-perceived healthcare climate, survey among 104 women after their prenatal consultation as part of a quantitative observation study in maternity care in the Netherlands, 2020 (N=104)

		*Not applicable n (%)*	*Strongly disagree n (%)*	*Somewhat disagree n (%)*	*Neutral n (%)*	*Somewhat agree n (%)*	*Strongly agree n (%)*
1	I feel that my maternity care professional offers me choices				1 (1.0)	5 (4.8)	98 (94.2)
2	I feel understood by my maternity care professional				2 (1.9)		102 (98.1)
3	I can be open with my maternity care professional during our appointments					2 (1.9)	102 (98.1)
4	My maternity care professional indicates that she has confidence in my ability to make choices regarding my pregnancy and childbirth	1 (1.0)			1 (1.0)	1 (1.0)	101 (97.0)
5	I feel that my maternity care professional accepts me						104 (100)
6	My maternity care professional has made sure that I really understand my options and the choices I have	4 (3.8)	1 (1.0)		3 (2.9)	5 (4.8)	91 (87.5)
7	My maternity care professional encourages me to ask questions	1 (1.0)	1 (1.0)	1 (1.0)	1 (1.0)	5 (4.8)	95 (91.2)
8	I have great confidence in my maternity care professional					1 (1.0)	103 (99.0)
9	My maternity care professional answers my questions completely and carefully	1 (1.0)		1 (1.0)		1 (1.0)	101 (97.0)
10	My maternity care professional listens to how I like to do things (regarding my pregnancy)	7 (6.7)			2 (1.9)	3 (2.9)	92 (88.5)
11	My maternity care professional is very good at dealing with people’s emotions	46 (44.2)		1 (1.0)	1 (1.0)		56 (53.8)
12	I feel that my maternity care professional cares about me as a person	2 (1.9)		1 (1.0)	1 (1.0)	8 (7.7)	92 (88.5)
13	I feel comfortable with the way my maternity care professional talks to me				1 (1.0)		103 (99.0)
14	My maternity care professional tries to understand how I see things before making a new proposal	5 (4.8)	1 (1.0)	2 (1.9)	2 (1.9)	4 (3.8)	90 (86.6)
15	I feel able to share my feelings with my maternity care professional	2 (1.9)			2 (1.9)	2 (1.9)	88 (84.6)

### Associations between professionals’ interactions and the woman-perceived healthcare climate

We found a weak but significant association between autonomy-supportive interactions and a positive woman-perceived healthcare climate (r=0.27, p=0.02). We found no association between structuring interactions and the woman-perceived healthcare climate (r=0.099, p=0.32).

The level of the woman-perceived healthcare climate was the same across the various individual professionals (p=0.370).

## DISCUSSION

This study aimed to quantify the frequency with which maternity care professionals use autonomy-supportive and autonomy-thwarting interactions during prenatal consultations in daily practice, and assess whether these interactions are associated with the woman’s perceived healthcare climate during the consultation.

The overarching finding of this observation study was that professionals base their autonomy-supportive interactions on a small repertoire. Moreover, they tend to use more autonomy-supportive interactions, giving room for the woman to participate, and fewer supportive interactions that stimulate active woman involvement. Regarding the structuring interactions, they tend to use more clarifying and informing interactions. Autonomy-thwarting interactions were hardly observed during regular prenatal consultations.

Although we found that different individual professionals exhibited different autonomy-supportive and structuring interactions, we found no differences between their consultations regarding the woman-perceived healthcare climate. There was only a weak significant association between the autonomy-supportive interactions and the woman-perceived healthcare climate.

The findings that professionals use informative interactions to meet women’s need for competence and mainly fulfil women’s need for autonomy by offering room to the woman are in line with the literature and as expected^2-6^. However, the more interesting finding of this study is that specific interactions were hardly observed, such as summarizing and asking the woman to repeat information. The lack of some interactions could have consequences for the consultation, as is further discussed in the following paragraphs. In line with our expectations, autonomy-thwarting interactions were hardly observed^16^.

The observation tool that is used categorizes interactions into need-supportive or need-thwarting interactions. Research in other domains, such as sports, suggested a third group of interactions between the need-supportive and need-thwarting interactions, the so-called need-unfulfillment interactions^23^. Based on this three-stage model, the woman could perceive need satisfaction, thwarting or unfulfillment. Need-unfulfillment is defined as the feeling that one’s need is neglected^23^. Maternity care professionals do not actively thwart women’s needs but could overlook them. Need-unfulfillment has been proposed to be related to more passive forms of functioning, such as women’s disengagement^24^. Looking at our results from the perspective of the proposed three-stage model, professionals seem to ignore some aspects of meeting women’s need for autonomy and competence by using a small repertoire of interactions, which could be perceived as overlooking them.

Focusing on some specific rarely observed interactions, we noticed that professionals rarely summarized their conversation or asked women to state what they understood of the discussed information in their own words. This so-called teach-back method is an evidence-based way of improving women’s understanding of the provided information, fulfilling their need for competence. Therefore, teaching back is part of almost every healthcare professional’s communication training^25^. Professionals rarely encourage women to consider possible approaches with their important kin. The importance of peer support for meeting women’s psychological needs and, thereby, supporting their more autonomous decision-making has been described in the literature^2,26^, not only from the perspective of meeting women’s need for autonomy but also from the perspective of meeting the other parent’s needs^27^. Studies have reported that the other parent could experience stress due to receiving insufficient information about pregnancy and childbirth^28^.

Other rarely observed interactions are intended to meet women’s need for autonomy, such as ‘asking for the preferred degree of autonomy’. Also, there were few observations of more structuring interactions to meet women’s need for competence, such as ‘stimulating self-reflection’ or ‘providing alternatives’. Professionals seem to struggle with how much they can or are allowed to support women’s competence, thereby supporting their more autonomous decision-making. However, the literature showed that professionals might offer structure and professional knowledge on the condition that women understand the importance of their contribution to the decision process, that women understand their options and the pros and cons, and that women’s views, concerns and preferences are included^29^. It is also essential to consider the degree of autonomy the woman desires^26^.

In general, women were satisfied with the perceived autonomy-supportive healthcare climate during consultations, which aligns with previous studies’ findings^30^. However, 44.2% of the women in our study indicated that dealing with emotions was not applicable during the prenatal consultation. To offer an autonomy-supportive healthcare climate and support women’s self-regulated behavior and more autonomous decision-making, professionals must become familiar with women’s emotions, such as fears, expectations, beliefs and motivations. Professionals become informed about these issues because women spontaneously share this information or because the professionals encourage the women to share this information^4,26^. However, when we compare the finding that dealing with emotions was not applicable during prenatal consultation with our observations of professionals’ interactions, we see that, in line with women’s perceptions, ‘allowing emotions and actively naming them’ was rarely observed. Discussing women’s emotions does not seem to be a standard part of a regular prenatal consultation.

The findings of our study provide insight not only into the interactions that professionals use but also into the effects the interactions could have on the conversations during prenatal consultations. Women were rarely stimulated to be actively engaged in the consultations. The effect of seldom-used interactions that stimulate active woman engagement could become even stronger because the need unfulfillment can cause more passive behavior and disengagement^23^. Women’s engagement is essential to offer tailored decision-making support^26,31,32^. In maternity care, this engagement is vital because knowing the woman helps make decisions about preferred care during birth^33^. Whether it is possible to realize the preferred care during birth depends on medical and organizational circumstances; it is essential that the professionals can help their women adjust their decisions during this process. While the woman is in the birthing process, it is more complicated to discuss what matters to them. Therefore, it seems crucial to have these conversations during prenatal consultations. Some women mentioned that the professionals did not know the woman’s situation, but knowing women’s fears, norms, and expectations is essential to meeting women’s need for autonomy. We could not provide an explanation for this finding based on our data. However, based on the observed warm climate during the consultations, we suggest that professionals sometimes unintentionally ignore women’s basic psychological needs. We suppose that they are not sufficiently aware of women’s basic psychological needs and the importance of meeting them.

Further research is needed to establish why professionals only partially meet women’s basic psychological needs. In the meantime, it could be helpful if professional training or continued professional training paid attention to the positive effects of meeting women’s psychological needs on women’s self-regulated behavior and, thereby, on their decision-making capabilities.

### Strength and limitations

A strength of our study is the detailed observation and coding of interactions between maternity care professionals and patients during prenatal consultations in daily practice.

It is also a strength that we included a representative sample of professionals who provide prenatal consultations in the Netherlands. However, this makes it impossible to say anything about the differences between the different groups of professionals. Also, the associations between individual professionals, the interactions they use, and the patient’s perceived healthcare climate should be taken with caution because of the small number of patients.

Since this research was not a longitudinal study, we do not know if some participating professionals and women were already so familiar with each other that they no longer needed to discuss specific issues. We suppose this possibility may have had a small effect on our results since professionals who already knew a woman well could align with this woman’s perspective without further questioning. This degree of familiarity was not observed frequently.

Various issues were discussed in the consultations, and different decisions were made. In the literature, some authors suggest that professionals vary their autonomy support based on the complexity of the decisions that must be made. Braddock et al.^34^ categorized decisions into basic, intermediate and complex, indicating that professionals used more autonomy-supportive behavior to make complex decisions. Based on these categories, most of the included consultations involved basic decisions (e.g. the timing of the next appointment) and complex decisions (e.g. induction of labor)^34^. Therefore, we assume that our results provide a representative reflection of autonomy-support during prenatal consultations.

The HCCQ assesses the perceived social context (i.e. the need-supportive climate) rather than assessing if women feel that their needs are supported (need satisfaction)^20^. Perceiving a healthcare climate as need-supportive does not automatically mean that the woman feels autonomous and competent. Also, the results may have been affected by the fact that women completed the questionnaire immediately after the consultation. Women may perceive the healthcare climate as less autonomy-supportive later on when they have time to reflect on the consultation. Although the consultations were not explicitly chosen and almost all the invited agreed to participate, there was an overrepresentation of women with a high level of education. This could affect the generalizability of our results. Women’s experiences while giving birth can influence their perceptions of the prenatal care they receive. For further research, it would be interesting to assess how women perceive the prenatal healthcare climate and decision-making process after giving birth.

## CONCLUSIONS

Maternity care professionals use interactions which support women’s need for autonomy by offering room and choice. They use interactions that provide information to meet women’s need for competence. As professionals are less inclined to use interactions that stimulate active women’s participation, it is harder for them to become acquainted with women, hindering them from offering tailor-made decision-making support and decreasing women’s autonomy. Also, women’s need for competence is only partly met because professionals do not stimulate women to discuss their deliberation process. The present study highlights not only the importance of meeting patients’ needs, but also the impact of neglecting them. To help professionals optimize their autonomy support, future research should investigate why professionals use specific interactions but avoid others. Professionals could improve their autonomy-supportive consultation practice by explicitly paying attention to interactions involving women and offering structure. More structured interactions may elicit active engagement, autonomy, and self-regulation, facilitating women’s decision-making.

## Supplementary Material



## Data Availability

The data supporting this research are available from the authors on reasonable request.
