# Supplementary material for: Autonomy support in prenatal consultation: A quantitative observation study in maternity care
Source: Eur J Midwifery. 2025 Jan 13;9:10.18332/ejm/197053. doi: 10.18332/ejm/197053 (PMC11726625; doi:10.18332/ejm/197053)
Supplement: Supplementary file 1 [file EJM-9-03-s1.pdf]

Supplemntary file

	TABLE 1		
**Item**		**Description**	**Example Maternity Care**
	**AUTONOMY-SUPPORT**		
	**Attuning approach**<br><br>The extent to which the professional starts from the woman's perspective		
	The Maternity care professional…		
1	…leaves room to the woman to tell	The professional leaves room for the woman to discuss her world and lifestyle. Takes time to get to know the woman's world. Both the woman and the professional can initiate the theme.	"How are you now?" The professional asks, and then the woman is given some time to think and answer (professional-initiated). Woman: "I am quite scared." The professional responds, "Tell me…" (woman-initiated).
2	…listens reflectively and explores	The professional listens reflectively during the conversation and, in this way, explores what is going on with the woman. The professionals see the feelings and thoughts of the woman that they recognizes. The professional connects with what the woman brings to the conversation and asks further questions.	"I hear you say that you are worried. Can you tell me more about that?<br>Or I hear you say that you find it difficult. What is the reason it is so difficult? Why do you think that way? How was it to get the results? What did that do to you?"
3	… aligns with the woman's perspective	The professional starts from the woman's perspective by investigating the difficulties and concerns the woman experiences in achieving goals or following advice and taking them into account.<br><br>The professional knows who she is dealing with, is aware of the personal living circumstances, or informs herself about them. (does not have to be directly related to obstetrics)	"What is it like for you to make a choice about…?<br>What makes it so hard to make this choice?"<br>"What would you need? What do you need?"
4	…uses questions that offer space to the woman	The professional uses open questions or questions that offer space to the woman. This item is rather professional-initiated. In item 1, the theme can be initiated by both.<br>Let the woman think about taking action. Questions encourage the woman to take action.	"What makes it so difficult to decide whether or not to induce at 37 weeks because of your abnormal liver function values?<br>What can I do to help you with this?<br>What do you need from me to be able to make a choice?"
5	…allows emotions and actively names them	The professional allows emotions (negative or positive) or reactions that indicate resistance in the woman and also actively names them.<br><br>Emotional reflection by professionals: the nature of the feeling is shown, and the intensity of the emotion.	"I get the impression that you find it difficult to choose because choosing the NIPT could have the consequence that you have to think about terminating your pregnancy. That idea affects you. Is that right?"<br>"I understand you are very worried about the delivery, is that right?"

6	…uses inviting language	The professional uses inviting language, using words such as 'can' and 'try' instead of 'must'.	What could help you with your morning nausea?<br>What would you like to do yourself?<br>"Is it possible to discuss the dilemmas you experience when choosing prenatal screening with someone close to you? Would that be helpful?"
	**Participative approach**<br><br>The extent to which the professional places the woman's agenda centrally and provides direction together with the woman		
**7**	…allows time	The professional gives space and time to come to a solution and explore.<br><br>Gives space to come to a decision. Decision-related (decision can be very small or big).	"Do not immediately fill in the silence yourself. Leave silence to let the woman answer, think for herself, pause for a moment, and let her come to a choice/question herself."
8	…gives voice	The professional offers the woman a say in dealing with the condition. This item also scores high if the woman continues to speak or suggests topics of conversation	"What do you want to know about the options for prenatal screening?"<br>"How would you like to discuss the different test options?"<br>"What information do you need from me to make a decision?"<br>"What do you need from me to make a choice?" "Would you mind if I gave you some additional explanation?"
9	…explores the woman's goals	The professional explores the woman's goals. This item also scores<br>high if the woman herself introduces/initiates goals.<br><br>Naming: requests for help, wishes, expectations.	"What is the purpose of prenatal screening for you?"<br>"What is most important to you during your pregnancy?"<br>"What is very important to you as we approach the birth?"
10	…encourages to think about a possible approach	The professional encourages the woman to think about a possible approach and works with the woman to approach the goals that the woman has set. An open intervention that stimulates thinking scores higher than a directive intervention. See examples [1] versus [2].	"What can help you choose prenatal screening or the method of delivery? "How do you see that in concrete terms?" [1]<br>"What can I do to help you achieve your goal?" [2]<br><br>Goals can also be good experiences despite the fact that they go differently. Discuss this in the birth plan. For example, what would you like if it unexpectedly becomes a caesarean section? What don't you want?
10a	**…**encourages to think about a possible approach with important kin	The professional encourages the woman to think together with important partners (family, friends and peers) about possible	Have you discussed the options for prenatal screening with your partner/family?

		approaches and possibilities. The peers support the woman in this from their connection with the woman.<br>(collective autonomy)<br>Regardless of any presence during the consultation	How does your partner/family feel about your participation in prenatal screening/home birth/breastfeeding?<br><br>What does your husband/partner think about it?<br><br>Have you discussed it together?
11	… offers an explanation	The professional offers the woman an explanation, statement, and rationale for making agreements or carrying out specific tasks or assignments. Here, the professional explains it herself.<br>In item 24 (stimulates self-reflection), the professional lets the woman think about a possible underlying explanation.<br>In item 29, the professional only provides information or instruction; there is no discussion of how something comes about or why something is important …<br><br>Explain, clarify, interpret, not inform (then score in item 29), how does it come about…<br>Explanation in echo or fundal height is informing.	“The baby is in breech position, which is less favourable to be born. Now, there are several possibilities. We can try to perform an external version at 36 weeks. You can also choose to do it…<br>”Smoking harms your placenta. It is less well supplied with blood due to smoking, which means the baby remains smaller.
12	… actively gauges what degree of autonomy the woman wants	The professional actively (explicitly and on her initiative) assesses the degree of autonomy the woman wants and can assume and which role the woman wants to assume in managing his health condition. This involves explicitly addressing it. It clarifies which role the woman wants to assume, how much control a woman wants to take in her care/in aspects of living with the condition/in decision-making	The assessment of motivation is not included here (item 22)<br>This is about the extent to which the woman wants to play an active role. The woman uses medication with possible consequences for the pregnancy and child, agreeing whether the woman wants to investigate this themselves or by the professional<br>Do you already know something about it?<br><br>Discussing smoking/prenatal screening or the woman indicates that they do not need an explanation.<br><br>For example, we can complete the birth plan together, and you can also complete it yourself.<br>Also, explicitly include matters in the birth plan, e.g. I do not want to be counselled about pain relief during labour.<br><br>It can also be general: During pregnancy, you are confronted with many choices; how do you like making choices? In what way do you like to make your choices?
	**CONTROL**		
	**Demanding approach**<br><br>The extent to which the professional starts from a health perspective and wants to keep control		
13	…shows expertise, demands respect	The professional shows his expertise by interrupting or correcting the woman and demanding respect. This can be present both verbally and nonverbally. Also, pay attention to the	“Waiting is not possible now; I want you now…”<br>Or even more compelling: you must<br>“It will not be easy to find a professional who wants to guide your delivery how you want. We realise that there are risks

		professional's attitude. The professional places herself above the woman.<br><br>Attitude of non-equality	associated with your delivery due to your medical indications, and we feel responsible for your health and that of the baby." "I know from experience that you will regret this choice"<br>Listen, I have a lot of experience with this. There is no time to explain everything now; you have to trust me.<br>Just trust me.
14	…uses controlling language	The professional orders the woman uses controlling language or often uses imperatives.<br><br>Closed questions and controlling verbs	I will reserve a date for your planned caesarean section in the planning because of the breech position/caesarean section in the history."<br>"I want you to go to the hospital now to have an CTG"<br>Or<br>"You are going to the hospital now to have an CTG.<br>I want you to go to the hospital now for an extra check-up/come to me.<br>I think you should rest more. From now on, you must rest for 1 1/2 hours every afternoon. I want Yo to stop working.
15	…takes over the conversation	The professional does or says things during the conversation and takes over the conversation from the woman. The professional also continues to provide explanations and interpretations without giving the woman the space to go into more depth. The professional generally outlines the course of specific diseases and does not start from the woman's situation. Item 17 (interrupts the woman) disregards the contribution that a woman is trying to make in the conversation.	"Most clients decide to have an ultrasound; the chance of finding an abnormality is small, so just have it done"<br>The "yes but" in response to an initiative/idea from the woman<br><br>"You must have been very shocked."
16	…determines the topics of the conversation	The professional determines the agenda and which themes are discussed or imposes her own preference.<br><br>Professional determines the agenda<br><br>Score frequency with which the professional determines the themes within the unit. If the professional maintains the theme for the entire unit, you also score this high. After all, this shows that professionals do not leave room for theme changes.	"You need to lie down on the couch first…"<br>I want to know how you are doing first. Then, I would like to complete your file and discuss the birth plan.<br><br>You are now 34 weeks pregnant; today, the birth plan discussion is on the agenda.<br>"I wanted to…" "Then you can lie down on the couch now."
17	…. interrupts the woman	The professional interrupts the woman and does not let her finish speaking, disregarding the contribution that a woman is trying to make in the conversation (compared to item 15).	Yes, but without an example.<br>I want to go back to..<br>I'm measuring your blood pressure in the meantime…
	**Domineering approach**<br><br>The extent to which the professional focuses on her own agenda, and there is pressure where the woman is attacked 'as a person'		
18	…puts pressure on the woman	. The professional pressures the woman to meet certain deadlines or make decisions by pointing out the negative consequences or complications of not following through on goals and uses (negative) results to put pressure on the woman	"You have to decide today or tomorrow whether you want to have a 20-week ultrasound." "If you do not agree with our obesity policy, we cannot supervise your pregnancy here, and it is better to find another practice." "If you cannot

			give birth on the ground floor, you cannot give birth at home."<br>You have to make a decision now; time is running out."
19	… expresses criticism	The professional gives negative, destructive criticism to the woman if she does not act as expected of her. There is a negative value judgment in the message, the person is played, this in contrast to item 23 (gives feedback)	"It is a risk that you take by going for a vaginal birth". "By not wanting to know in advance, you and we cannot prepare for possible complications due to a condition in your child."<br><br>This can also be seen in non-verbal expressions, for example shaking your head, or short expressions like "ah, so"<br><br>We agreed that you would stop smoking, but now it turns out that you smoke 3 cigarettes a day<br><br>Waiting after 42 weeks of pregnancy is really very unwise. It is very unwise to want to give birth after a caesarean section without a CTG.
20	…introduces guilt and shame	The professional puts pressure on the woman by appealing to her sense of self-worth or pride. He plays on feelings of guilt and shame to stimulate the woman; this can also be done by making inappropriate social comparisons.	Smoking is also a good example here: Didn't you quit? Do you know how bad it is for the baby?<br><br>You are taking a risk by waiting for the natural course and not inducing it. Your child could die.<br><br>Bring up BMI every time. With your weight, you can't give birth at home or in a bath.<br><br>"Don't you have your child's best interests at heart?"
21	…is irritated, imwoman	The professional is irritated and loses patience.	Sighing, intonation, tapping with a ballpoint pen/nails, looking at the watch, getting up,…<br><br>Rushing: taking anamnesis while doing a physical examination, research during the interview, little room for questions (making up for time)
	**STRUCTURE**		
	**Guiding approach**<br><br>The extent to which the professional provides support that makes the woman more competent		
22	...sets realistic goals in collaboration	The professional sets feasible, realistic (Interim) goals together with the woman.	"You have received a lot of information today; when do you think you can make a choice?"

		The professional formulates the goal, and the woman adopts it, ensuring the formulation aligns with the woman's wishes. (max 2 score)	“Do you think you can make a decision yourself or do you need support?”<br>“How many fewer cigarettes would you like to smoke in the coming week?”<br><br>“I think it would be wise to …., Yes, you are right, I need to make a change and think more about myself.”
23	…provides task-oriented or progress-oriented feedback	The professional offers task-oriented feedback on things the woman has done and behaviour she has displayed. This can be done<br>based on action points and tips. The professional gives positive and progression-oriented feedback to motivate the woman. It is about current affairs, about the here and now. Is formulated neutrally, the woman makes progress with the message, in contrast to item 19 (gives criticism) or item 34 (inappropriate feedback)<br>Feedback should work towards strengthening the pregnant woman's competence (being competent). Merely informing about physiological progression does not fall under this (e.g. your blood pressure is good' = item 29)	“I notice that you have prepared well for this consultation”.<br>“It is good that you have written down all the questions following our previous conversation”.<br>Compliment the woman. “I think it is great that you have solved all the problems with the housing association so well.”<br>.<br><br>“
24	…stimulates self-reflection	The professional stimulates the woman's self-insight through self-reflection. Here, the professional lets the woman think (in item 11, the professional explains it herself).	“What makes it so hard to make a choice?” “What could help you make a decision?” “Why do you prefer not to think about your delivery?”
25	…mentions previous successes	The professional refers to situations where the woman has previously had successful experiences. This concerns previously stated behavior and may occur more in situations of relapse. (item 23 concerns current behavior)<br><br>When referring to a previous successful experience, specifically mention it. If not, give positive feedback (then score at item 23)	“Last time, you also managed to get a good OGTT despite your fear of needles.”<br>“Last time, you also did very well with the vaginal ultrasound.”<br>“You have already reduced smoking; that last step will also work.”<br>“You have stopped smoking before; what made it work then?”<br>“You gave birth once before, and then with Luuk, everything also went very smoothly/well.”
26	.... uses tools	The professional uses tools to clarify the verbal information. When these tools are used as clarification during the conversation, this scores higher. However, when a brochure, diagram, or curve is given to read at home without going over it during the conversation, this scores ‘1’.<br><br>Use of woman’s abdomen and ultrasound image as a tool for verbal explanation<br><br>Do not only use centimetres but also explain value and meaning.<br><br>Only referring to information (site/folder) is letting the women figure it out for themselves.<br>(score under item 38)	 Use of prenatal screening flowcharts, information brochures, and plastic models/drawings. Look up information on the PC and use it in the conversation to clarify matters to the woman; the professional points out specific muscles/organs to clarify a mechanism.<br>Point out development on a poster.

	**Clarifying approach**<br><br>The extent to which the professional provides clarification and communicates expectations to the woman		
27	…provides alternatives	. The professional provides an overview of the various options/alternatives for the woman.	“In addition to the NIPT, there is also the option of the combination test or just the ultrasound at 20 weeks”.<br><br>Everything is fine. We usually make a new appointment for 3 weeks, but if you prefer to return after 2 or 4 weeks, that is also fine.<br><br>In addition to inducing, you can also have a CTG made every day and wait.<br>There are more forms of pain relief, such as warm water (shower/bath); you can give birth in many different positions, such as standing or squatting…
28	…uses appropriate role models	The professional uses appropriate examples of other women as role models with whom the woman can identify<br><br>Closer than a general reference group, e.g. priming, must be personal.<br><br>Must be recognizable and reinforcing/positive	“You are not the only one who finds it difficult to decide. Many pregnant women find it a difficult choice, but in the end, everyone manages to make a decision.”<br><br>Referring to own mother, sister, friend
29	…provides information	The professional provides clear and concise (oral) information.	“A child is born with Down syndrome. It is a condition that will not go away. People with Down syndrome have an intellectual disability and often look different. It is impossible to say in advance how serious the disability will be.”<br>Information about pain management and the advantages and disadvantages of the different forms.<br>Information about home birth and breastfeeding..
30	…summarizes and requests repetition	The professional summarises what was said and repeats or asks the woman to repeat the information given briefly.<br>It is essential to check whether the conversation leads to clarification for the woman. The more components (summarising, repeating, having<br>repeated), the higher the score. Only summarising scores a '1'. When the professional effectively asks the woman to repeat the information briefly, this scores a '4'.	“We just discussed the possibilities for prenatal screening at the beginning of pregnancy. Can you repeat for me what the most important differences are between the combination test and the NIPT test”.<br>“What do you get out of it as the most important difference.”<br>“Can you say…<br>Can you summarise what I said to see if I have been clear?”<br>“What are the most important differences between an epidural and remifentanil for you.”
31	...clarifies follow-up of women’s goals	. The professional explains how the goals set will be followed up.	“I am now going on maternity leave for four months. I will write down what we discussed and which questions are still open. My colleagues will get back to you about that.”

			"You will come to my colleague next week for the ultrasound and then we will see each other again in 4 weeks."<br>"If you have decided before the next appointment, you can also inform me by phone so I can prepare the paperwork for you."<br>"Next time we talk about the delivery, perhaps your partner would like to come along."<br>"You will come to my colleague next week for the ultrasound, who will also discuss the results and what you want to do with them. Of course, I am also available for questions."
…<br>32.	…uses attuned language	The professional uses language that is adapted to the woman's level, language that is understandable to the woman. The professional avoids jargon.<br><br>Only avoiding jargon scores max 3.<br>Really trying to connect with the woman's language and choice of words is 4.	.
	**CHAOS**		
	**Abandoning approach**<br>The extent to which the professional lets things run their course and no longer intervenes.		
33	…provides information that leaves woman in uncertainty	The professional provides unclear, incorrect or ambiguous information about the behavior that the woman is performing or is expected to perform. It is not clear to the woman what she should do.	Woman response: "Now you say that in case of a breech presentation, a caesarean section is performed in principle. Last time you said that, in consultation with me, a choice is made based on.."<br>Woman response: "You are now saying that I have to make a decision today, but the brochure says..".<br><br>Also, giving an overload of information<br>A lot of information, nothing to do with it<br><br>But last time, they said that an ultrasound would be performed today
34	…gives inappropriate feedback	The professional gives inappropriate/unjustified feedback about the behavior or approach that the woman proposes (process or action-oriented). This form of feedback is of little or no help to the woman, the feedback creates uncertainty or doubt in the woman (in contrast to item 23 "gives task or progress-oriented feedback")	"How can you decide if you haven't even read the brochures?"<br>"How can you make a good choice if you haven't filled out the decision aid?"<br>"If you were my partner, I would!"
35	…uses an illogical conversation structure	The professional uses an illogical, incoherent structure during the conversation.	The professional jumps from one topic to another and often has to return to<br>clarify matters or terms.<br>The professional does not clearly provide an overview of the possibilities for prenatal screening. He mixes up the 20-week ultrasound and screening at the beginning of pregnancy.

			Information at the wrong times:<br>At 6 weeks, triplets are discovered on the ultrasound and then immediately start talking about reduction.<br>At 12 weeks, start talking about the delivery.<br>At 40-year-old prim, indicate at the beginning of pregnancy that she will never reach 40 weeks.
36	…ignores reactions or concerns	The professional ignores the woman's reactions, both verbally and nonverbally. The professional nonchalantly dismisses the woman's expressed concerns instead of exploring them further and determining where the woman's concerns come from.	The professional does not respond to concerns/questions that the woman expresses verbally. The<br>woman sighed deeply during the conversation, but the professional<br>didn't ask where the sigh came from or what it caused<br>"Don't worry now; millions of women have already given birth to a child."<br>"You are worrying unnecessarily".<br>It always turns out fine.<br>Not one child stays behind; they all come out.<br>Not one has ever stayed behind.<br><br>"Do not respond to the woman's comment.<br>I drank a lot once at the beginning of the pregnancy; that was to celebrate the negative test"
	**Awaiting approach**<br><br>The extent to which the professional offers little or no support to the woman		
37	…does not intervene	The professional allows the woman to continue her behaviour or choices and does not intervene.<br><br>Missing/not picking up a signal	"It's your choice; if that's what you want, I'm fine with it."<br>"You have to decide for yourself. it's your delivery/pregnancy"<br>It's unwise to go home with bleeding during pregnancy against our advice, but if you want to.<br><br>If you don't want to stop smoking, that's your choice.

			If you don't want to be induced<br>"We'll see how it goes."
38	…lets the woman find out for	The professional lets the woman figure it out for themselves in all areas.<br>This is about the lack of guidance and coaching by the professional. The problem is not solved together with the woman.<br><br>Leaving the choice to the woman without providing a tool, information, or guidance to make the choice.	If the woman keeps eating too much and gains too much weight, no more feedback on the weight<br><br>Here is a brochure for you<br><br>Let me know what you have chosen.
38	…is distracted, absent	The professional is busy with other things, is absent during the conversation,<br>And has little attention for the woman. When the professional is distracted by an external factor, this should also be scored high.	The professional is busy at the computer, looking at the computer screen instead of the woman, looking up documents during the conversation, answering the phone when it rings, and being distracted by a colleague who comes in for a quick question…

Supplemntary file

Figure 1: Descriptives of the COUNSEL-CCE axes, subfactors and indicators

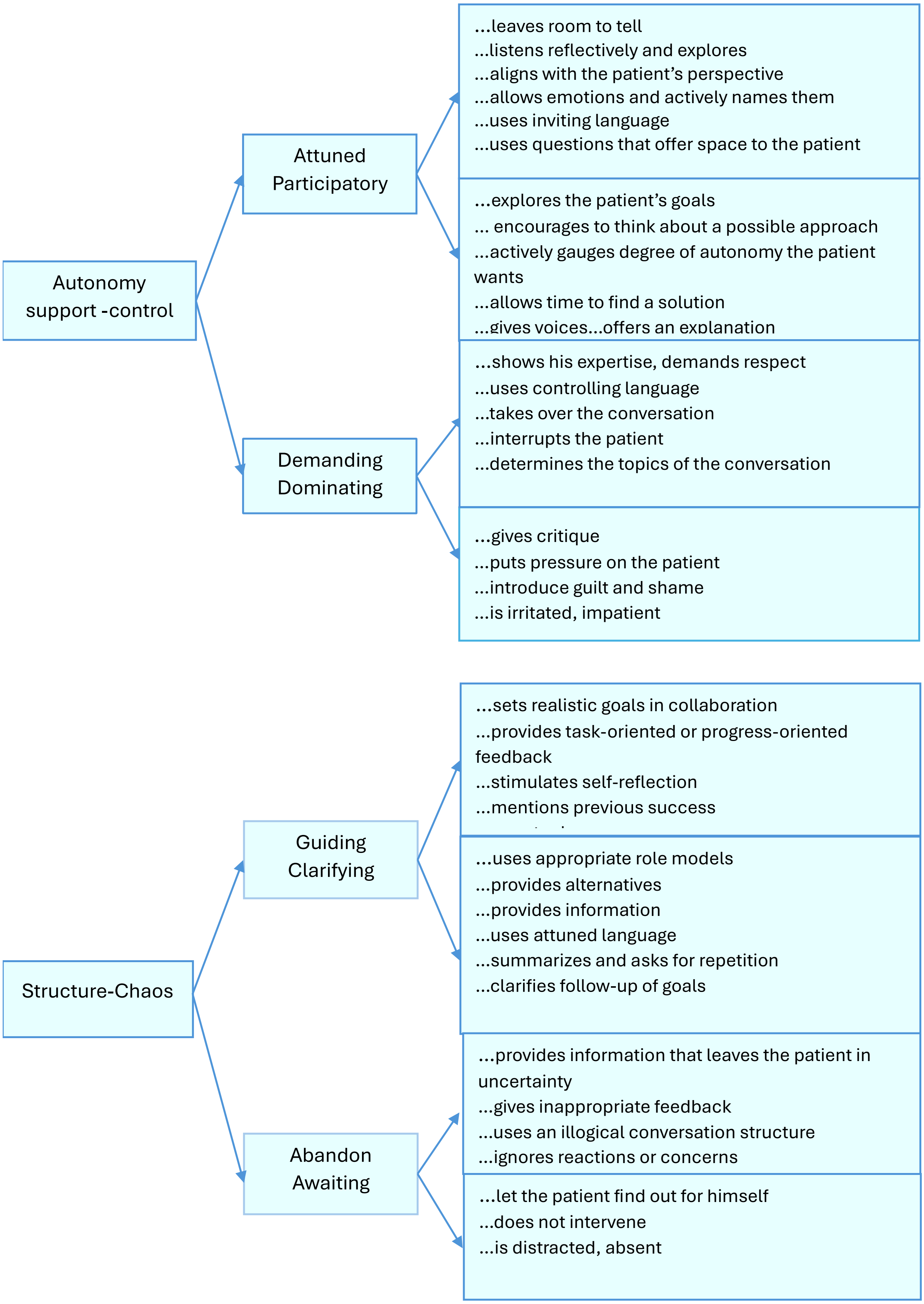

Supplemntary file

Table 2: Nederlandse Zorgklimaat vragenlijst (HCCQ) (Dutch HealthCare Climate Questionnaire)

Deze vragenlijst bestaat uit stellingen die gaan over uw verloskundig zorgverlener in het voorafgaande consult. Verschillende zorgverleners hebben verschillende stijlen in hoe ze omgaan met patiënten/ cliënten, en we willen graag weten hoe uw ervaringen zijn met uw verloskundig zorgverlener. Uw antwoorden zijn vertrouwelijk en zullen niet besproken worden met uw zorgverlener. Antwoordt u alstublieft eerlijk.

Omcirkel een cijfer van **1 (helemaal niet mee eens)** tot **7 (helemaal mee eens)** achter elke stelling.

Code audio-opname		Beoordelaar	
tijdslot		Code pate	

**1**	**2**	**3**	**4**	**5**	**6**	**7**

1. Ik heb het gevoel dat mijn verloskundige/ gynaecoloog mij keuzes biedt.	**1**	**2**	**3**	**4**	**5**	**6**	**7**
2. Ik voel mij begrepen door mijn verloskundige/ gynaecoloog.	**1**	**2**	**3**	**4**	**5**	**6**	**7**
3. Ik kan open zijn naar mijn verloskundige/ gynaecoloog tijdens onze afspraken.	**1**	**2**	**3**	**4**	**5**	**6**	**7**
4. Mijn verloskundige/ gynaecoloog geeft aan dat zij vertrouwen heeft in mijn vermogen om keuzes te maken met betrekking tot mijn zwangerschap en bevalling.	**1**	**2**	**3**	**4**	**5**	**6**	**7**
5. Ik heb het gevoel dat mijn verloskundige/ gynaecoloog mij accepteert	**1**	**2**	**3**	**4**	**5**	**6**	**7**
6. Mijn verloskundige/ gynaecoloog heeft ervoor gezorgd dat ik mijn mogelijkheden echt begrijp en welke keuzes ik heb	**1**	**2**	**3**	**4**	**5**	**6**	**7**
7. Mijn verloskundige/ gynaecoloog moedigt mij aan om vragen te stellen	**1**	**2**	**3**	**4**	**5**	**6**	**7**
8. Ik heb veel vertrouwen in mijn verloskundige/ gynaecoloog	**1**	**2**	**3**	**4**	**5**	**6**	**7**
9. Mijn verloskundige/ gynaecoloog beantwoordt mijn vragen volledig en zorgvuldig.	**1**	**2**	**3**	**4**	**5**	**6**	**7**
10. Mijn verloskundige/ gynaecoloog luistert naar hoe ik graag dingen doe. (met betrekking tot mijn zwangerschap)	**1**	**2**	**3**	**4**	**5**	**6**	**7**
11. Mijn verloskundige/ gynaecoloog kan erg goed omgaan met emoties van mensen.	**1**	**2**	**3**	**4**	**5**	**6**	**7**

12. Ik heb het gevoel dat mijn verloskundige/ gynaecoloog om mij geeft als persoon	**1**	**2**	**3**	**4**	**5**	**6**	**7**
13. Ik voel mij niet goed bij de manier waarop mijn verloskundige/ gynaecoloog tegen me praat	**1**	**2**	**3**	**4**	**5**	**6**	**7**
14. Mijn verloskundige/ gynaecoloog probeert te begrijpen hoe ik dingen zie voordat zij een nieuw voorstel doet.	**1**	**2**	**3**	**4**	**5**	**6**	**7**
15. Ik voel mij in staat om mijn gevoelens te delen met mijn verloskundige/ gynaecoloog	**1**	**2**	**3**	**4**	**5**	**6**	**7**

© 2024 Kors J. et al.
